# Hyperpolarised [2-^13^C]-pyruvate by ^13^C SABRE in an acetone/water mixture†

**DOI:** 10.1039/d4an01005a

**Published:** 2024-10-21

**Authors:** Oksana A. Bondar, Gamal A. I. Moustafa, Thomas B. R. Robertson

**Affiliations:** a School of Chemistry, Highfield Campus Southampton SO17 1BJ UK t.b.r.robertson@soton.ac.uk; b ATDBio (Now Part of Biotage), Highfield Campus Southampton SO17 1BJ UK

## Abstract

Signal Amplification By Reversible Exchange (SABRE) can provide strong signal enhancement (SE) to an array of molecules through reversible exchange of parahydrogen (pH_2_) derived hydrides and a suitable substrate coordinated to a transition metal. Among the substrates that can be used as a probe for hyperpolarised NMR and MRI, pyruvate has gained much attention. SABRE can hyperpolarise pyruvate in a low cost, fast, and reversible fashion that does not involve technologically demanding equipment. Most SABRE polarization studies have been done using methanol-d_4_ as a solvent, which is not suitable for *in vivo* application. The main goal of this work was to obtain hyperpolarized pyruvate in a solvent other than methanol which may open the door to further purification steps and enable a method to polarize pyruvate in water in future. This work demonstrates hyperpolarization of the [2-^13^C]pyruvate as well as [1-^13^C]pyruvate by SABRE in an acetone/water solvent system at room temperature as an alternative to methanol, which is commonly used. NMR signals are detected using a 1.1 T benchtop NMR spectrometer. In this work we have primarily focused on the study of [2-^13^C]pyruvate and investigated the effect of catalyst concentration, DMSO presence and water *vs.* acetone solvent concentration on the signal enhancement. The relaxation times for [2-^13^C]-pyruvate solutions are reported in the hope of informing the development of future purification methods.

## Abstract 2

The NMR hyperpolarisation technique SABRE has been used to hyperpolarise ^13^C labelled pyruvate in a predominantly aqueous solution which has then been measured by benchtop NMR.

## Graphical abstract

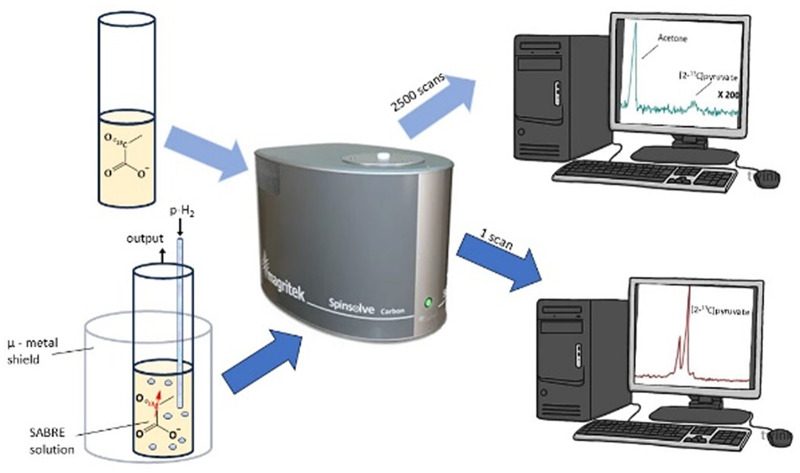

## 1 Introduction

**Fig. 1:**
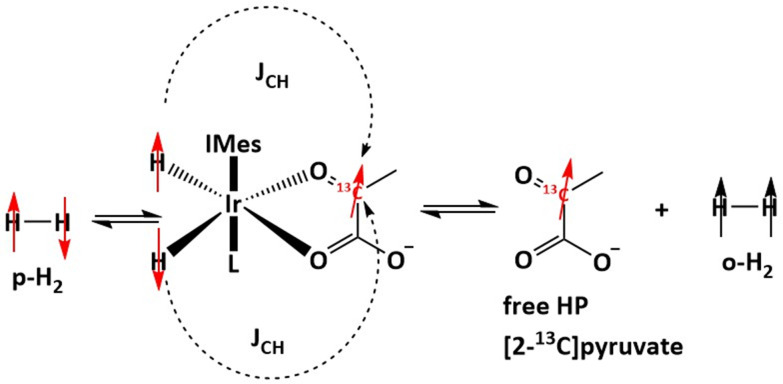
SABRE hyperpolarization of [2-^13^C]pyruvate, where L = DMSO/solvent.

Nuclear Magnetic Resonance (NMR) spectroscopy is a key analytical technique which may enable access to a wealth of information in a non-destructive manner. Unfortunately, NMR suffers from a lack of sensitivity which may lead to long acquisitions of many transients being required for sufficient signal to noise to be accumulated. As an alternative to long acquisition times hyperpolarization methods are now widely used to enable rapid high sensitivity experiments. One such field of hyperpolarization methods make use of parahydrogen (pH_2_), a spin isomer of hydrogen, which has been reviewed elsewhere.^1^ This parahydrogen may either be used to hydrogenate a suitable (*e.g.* unsaturated) substrate in a technique known as ParaHydrogen Induced Polarization (PHIP) or a suitable transition metal catalyst may be used to mediate polarisation transfer in the Signal Amplification By Reversible Exchange (SABRE) method.^2^ In SABRE (Fig. 1), polarization transfer from pH_2_ to ^13^C is mediated by the indirect dipolar *J*-couplings between nuclei in the transient complexes. Because of reversible interactions, a labile complex of pH_2_, catalyst and the ligand is formed. In this complex, high spin polarization is transferred from pH_2_ to the ligand *via* the catalyst.

As there is constant exchange of the ligand *trans* to parahydrogen derived hydrides (in addition to the hydrides themselves) this leads to a build-up of hyperpolarised substrate in solution. Regarding spin physics, many theoretical descriptions of polarization transfer to various nuclei at low^3^ and high^4^ magnetic fields have been presented on the example of various compounds and their isotopes. The spin dynamics depend obviously on the structure and chemistry of the SABRE complex. The structure of the SABRE complex has been discussed in several works.^5–7^ A vast majority of these studies are concerned with [Ir(COD)IMes]Cl, (IMes = 1,3-bis(2,4,6-trimethylphenyl)imidazol-2-ylidene; COD = cyclooctadiene) as a precatalyst, which forms a ternary labile octahedral complex after activation. The kinetics and thermodynamics have also been discussed.^8^ As SABRE may rapidly polarise a suitable substrate within seconds SABRE is a promising approach for hyperpolarizing substrates that have biological properties and many bio-relevant molecular systems have already been hyperpolarized.^5,9–14^ Several of these molecules are of central importance in MRI, where they can serve as the contrast agents.^15^ One of most promising and already widely used contrast agent in both pre-clinical and clinical trials is pyruvate.^16–20^ Since pyruvate is a natural metabolite that is converted to lactate by lactate dehydrogenase (LDH), it is completely harmless to the body. Hyperpolarized ^13^C pyruvate is widely used for a study of various types of cancer^16,19,21–25^ because it is known that cancer cells convert pyruvate to lactate much faster than healthy cells, therefore, using MRI technology, different types of cancer can be studied and diagnosed. Most research show metabolism of HP [1-^13^C]pyruvate as a mostly used contrast agent,^20,26^ but recently the first hyperpolarized [2-^13^C]pyruvate MR studies of human brain metabolism have been reported.^27^ [2-^13^C]Pyruvate provides new metabolic information distinct from HP[1-^13^C]pyruvate, because of [2-^13^C]pyruvate conversion to [2-^13^C]lactate and [5-^13^C]glutamate. As has been previously reported SABRE can hyperpolarise pyruvate in a low cost, fast, and reversible fashion that does not involve technologically demanding equipment.^28^ Unfortunately several challenges remain before this technique may be safely applied *in vivo*, notably the presence of the solvent and the catalyst as these have previously been shown to be cytotoxic.^29^ While several approaches have been previously reported in an attempt to remove the catalyst from solution,^30–33^ as well as solvent traces.^34^ In this work we report SABRE in an acetone/water mixture which we hope may facilitate the rapid production of an aqueous ^13^C hyperpolarised pyruvate bolus *via* acetone removal. Another advantage of using acetone instead of methanol other than higher volatility (that can be very useful during purification step to perform it faster) is lower toxicity (ESI.I.†)

## 2 Materials and methods

All samples (Table 1) made use of 5 mm Young's capped NMR tubes (5 mm Precision NMR Sample Tube Low Pressure/Vacuum Valve (LPV) 8L, 500 MHz). In all cases the precatalyst has been activated by bubbling of 85% enriched pH_2_ (Fig. S2†) (Bruker parahydrogen generator) through the solution. Bubbling was performed in a μ-metal-shielded solenoid with an internal field of 9 mG at room temperature.

**Table 1:** Summary of samples used within this work

Number	[Pyruvate], mM	[Catalyst], mM	[DMSO], mM	*V*(D_2_O), μl	*V*(acetone-d_6_), μl	*V*(CD_3_OD), μl	SE
I	36	4	23			600	2662 ± 142
II	36 ([1-^13^C]pyruvate)	4	23	200	400		1266 ± 34
III	36	4	—	200	400		477 ± 54
IV	36	4	23	200	400		733 ± 20
V	66	0.35	8	100	200		51 ± 5
VI	38	0.18	8	(100 + 300)^a^	200		33 ± 2
VII	58	0.7	8	(50 + 300)^a^	100		53 ± 2
VIII	32	0.13	23	200	400		42 ± 7

a *n* + *m*, where *n* – starting volume and *m* – volume added after catalyst activation.

Reagents and solvents were purchased from standard suppliers and used without further purification. Hyperpolarized signals were acquired immediately after first set of bubbling (20 s) after 1 scan while thermal signal had been received after up to 2500 scans.

Signal enhancement calculation were carried out making use of the equation below. Spectra are automatically re-scaled to the number of scans within the acquisition software (SpinsolveExpert 2.01.08).
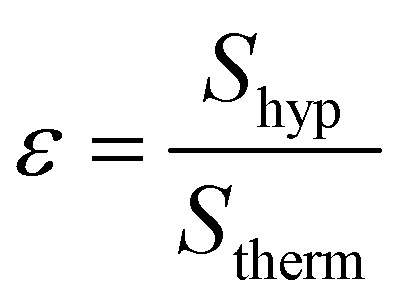
where *S*_hyp_ = signal integral from the hyperpolarised spectrum, *S*_therm_ = signal integral from unpolarized sample at thermal equilibrium.

### 2.1 Instrumentation

Hyperpolarized experiments (Fig. S1†) were carried out on a Spin-Solve 1.1 T benchtop NMR. Experiments for concentration confirmation, purity check and parahydrogen percentage measurements were performed on Bruker Avance Neo systems at 9.4 T and 16.45 T. For *T*_1_ relaxation measurement experiments these were carried out on the in the stray field of the 9.4 T magnet as previously reported.^35^ The samples were pre-polarized at 9.4 T and then transferred to the interference field regions. Sample transport was performed using a stepper motor driven sample cart operating at a fixed speed of 1 m s^−1^ as described previously.^36^ Signal detection was accomplished by moving the sample back into the detection region of the high-field NMR magnet and applying a π/2 pulse.

## 3 Results and discussion

### 3.1 ^13^C SABRE of [2-^13^C]pyruvate in methanol-d_4_

**Fig. 2:**
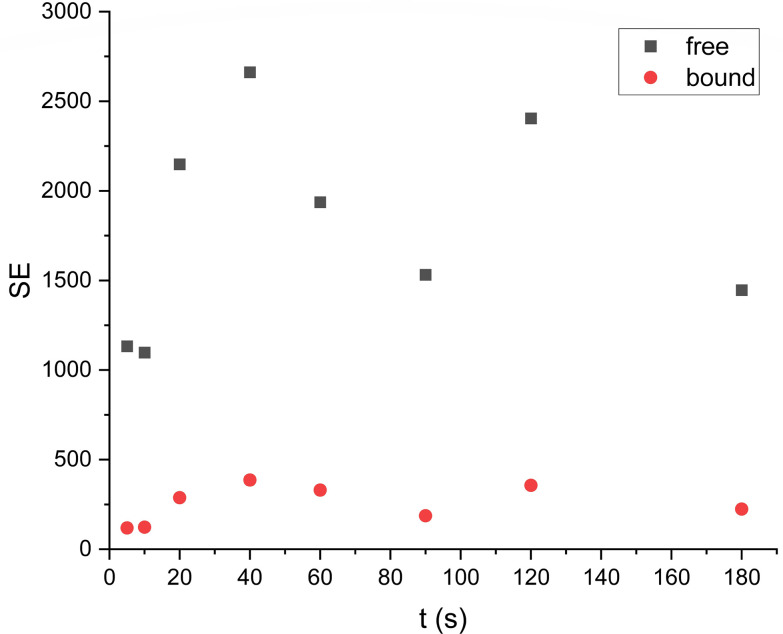
SE dependence for free and bound [2-^13^C]pyruvate sample I from bubbling time in methanol-d_4_ as solvent.

The majority of previous SABRE work has been undertaken within methanol-d_4_ therefore before moving onto a non-alcoholic solvent system we initially evaluated the ^13^C SABRE of [2-^13^C]pyruvate in methanol-d_4_. Making use of sample (I) high levels of ^13^C signal enhancement (SE) were observed and signals of pyruvate both free in solution and bound as part of the iridium complex were observed.^28^ As both free and bound signals were visible, the SE after a range of pH_2_ bubbling times were recorded, as shown in (Fig. 2, and ESI Table S1†). The highest level of SE was observed for free pyruvate after 40 s bubbling of pH_2_, SE = 2804. For this sample in methanol we can observe signals from both bound and free pyruvate while in the acetone/water mixture samples described below only the hyperpolarised signal from free pyruvate has been found, likely due to lower recorded signal enhancements. Free and bound pyruvate signals in the methanol solution were identified using previously published data.^37^ In the case of the acetone/water mixture the signal of acetone in D_2_O (Fig. S5†) was used as a chemical shift reference.

### 3.2 Effect of DMSO as a SABRE co-ligand in acetone/water for [2-^13^C]pyruvate

It has been previously reported that pyruvate had weak iridium pyruvate binding, which prevents typical hyperpolarisation of pyruvate using SABRE and that the presence of an appropriate dimethyl sulfoxide (DMSO) as a co-ligand allows for the assembly of a highly reactive polarisation transfer catalyst that overcomes poor pyruvate ligation to the iridium centre.^28^ It was reported by Iali *et al.*^28^ that DMSO as a co-ligand allows appropriate chemical lifetimes of complexes suitable for the polarization transfer from pH_2_ to pyruvate, where the ^13^C nucleus has small *J*-coupling values to the hydride protons in the complex.

**Fig. 3:**
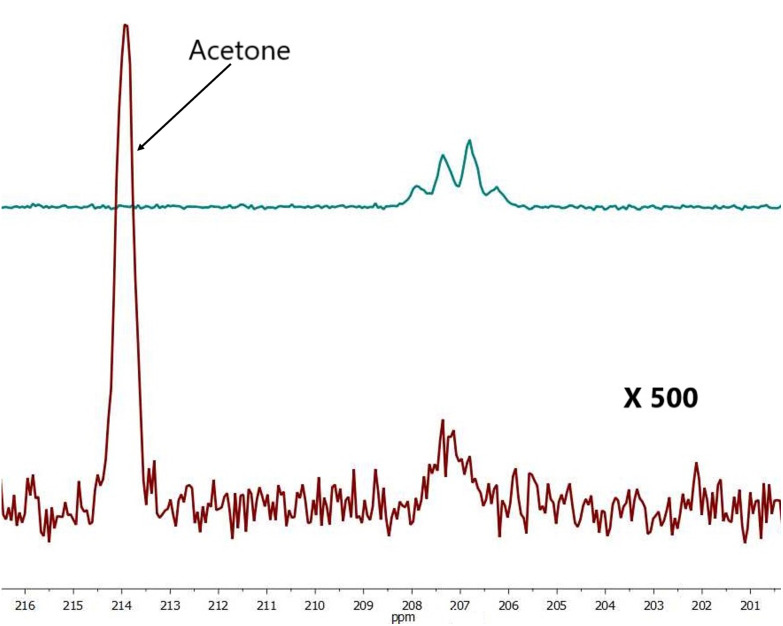
Comparison hyperpolarized (green) and thermal (red, 1256 scans ×500 times) spectra of sample III. For free pyruvate observed peak splitting is due to neighboring protons. SE = 437.

**Fig. 4:**
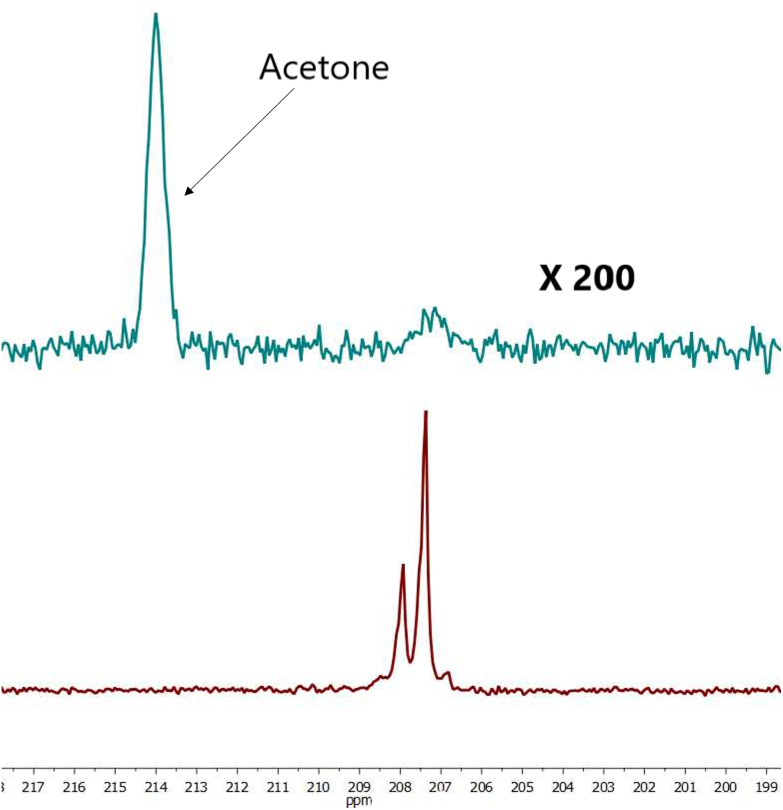
Comparison hyperpolarized (red) and thermal (green, 2500 scans ×200 times) spectra sample IV for free pyruvate observed peak splitting is due to neighboring protons. SE = 752.

To study the effect of DMSO for the acetone/water solution we performed two types of experiments: with- and without DMSO addition as a co-ligand. Data of the solution (III) hyperpolarized by SABRE conducted in the absence of DMSO resulted in SE for [2-^13^C]pyruvate of 477 ± 54 (Fig. 3), but significantly increased following DMSO addition (IV) (SE = 733 ± 20, Fig. 4). For sample (III) without DMSO as a co-ligand the signal enhancement is attenuated upon repeat experiments until no longer being observed around 1 hour following initial catalyst activation. This can be explained by catalyst deactivation. This is supported by qualitative observation that over time sample (III) without DMSO is observed to change colour from near-transparent yellow to dark brown on the timescale of hours, indicative of a change in iridium complex present. Whereas for sample (IV) with DMSO no colour change is observed over week long timescales. Solvent evaporation during p-H_2_ bubbling was checked (Fig. S9†) and found that a volume of 7 μL was lost over 20 s of bubbling time or 1.2% which we expect to have a minimal effect on solution concentration and SE.

**Fig. 5:**
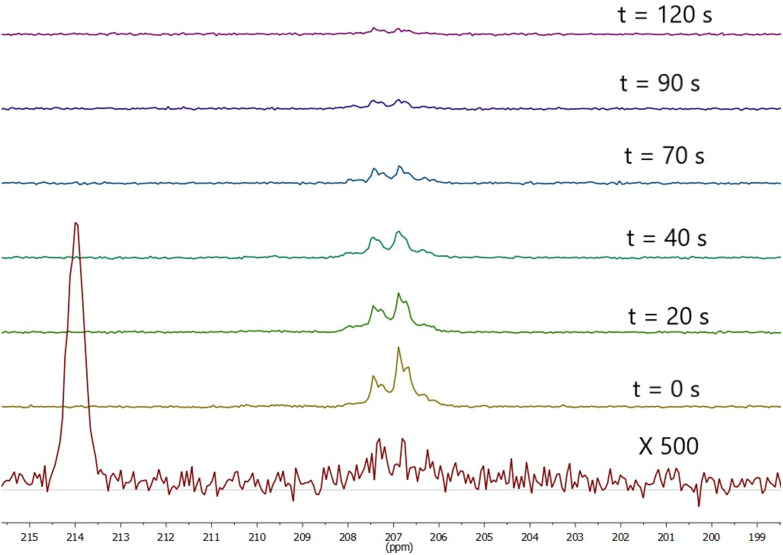
Observed signal at 1.1 T *t* seconds SABRE. SABRE was performed by bubbling for 20 seconds prior to *t* = 0 then stopping pH_2_ flow. Bottom spectrum is thermal polarisation at 1.1 T recorded after 1332 scans.

**Fig. 6:**
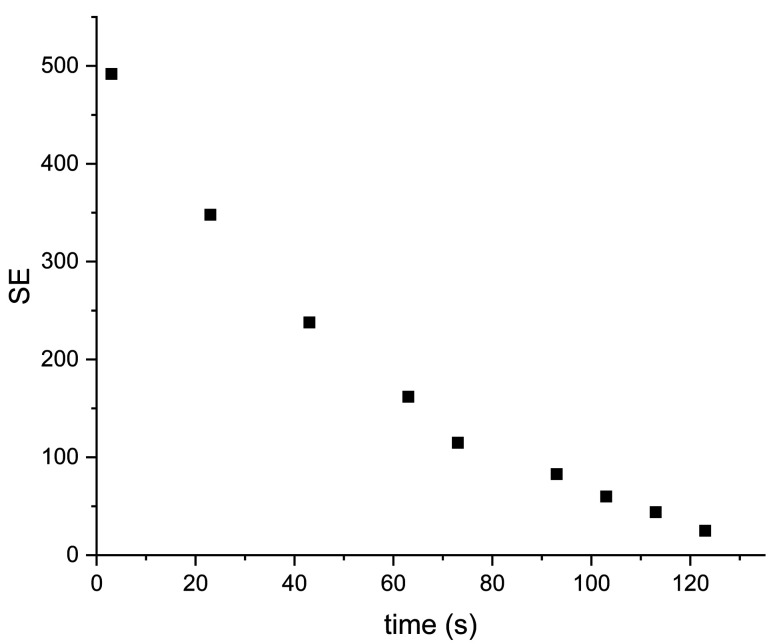
Decay of signal enhancement over time for sample IV at 1.1 T. *T*_1_ = 64 ± 3.5 s.

It was found the relaxation time under at 1.1 T was 64 s meaning that a hyperpolarised signal is still visible even after 2 minutes (Fig. 5 and 6) after the first measurement of the hyperpolarized sample, with the *T*_1_ measured at 1.1 T following SABRE measurement. This gives us strong confidence we can perform purification steps similar to ref. 13 with saving time for injection and measurements where time spent for purification steps was a round 55 s.

Based on measured *T*_1_ of [2-^13^C]pyruvate at the ref. 27 of 47 s and measurements done in our laboratory using a previously reported shuttle system, mounted on a 9.4 T magnet as described by Bengs *et al.*^35^ for [2-^13^C]pyruvate in aqueous solution in different fields (Fig. S7†) we can assume that it will be enough time for purification steps due to high volatility of acetone compare to methanol used in ref. 13.

In order to measure *T*_1_ for samples (III) and (IV) these were performed using shuttle system. It should be noted that changing the DMSO concentration did not effect either the measured SE or *T*_1_ for sample (IV). Comparison of *T*_1_ relaxation times could be observed at the Fig. S6.†

### 3.3 Effect of water on SABRE enhancement of [2-^13^C]pyruvate

To attain sufficient solubility for both the [Ir(IMes)(COD)Cl] pre-catalyst and the pyruvate substrate an acetone–water mixture was used as described in Table 1. Sodium pyruvate is essentially insoluble in pure acetone and the SABRE precatalyst is insoluble in water. A 2 : 1 ratio of acetone to water has been used herein.

Hyperpolarization of [2-^13^C]pyruvate by SABRE in acetone/water mixture with DMSO as co-ligand addition (IV) has been performed.

For sample (V) experiment concentrations of catalyst and DMSO were decreased as well as volume of the final solution, but still with ratio 1 : 2 D_2_O to acetone-d_6_. The same ratio of solvents, but different concentrations of pyruvate and catalyst have been used for further experiments with sample (V). The (V) solution was been polarized by SABRE with the highest SE = 55 after 90 s bubbling of parahydrogen through the solution. The next step was to polarize pyruvate in near aqueous solution. For that purpose sample similar to (V) was prepared, but after full activation of the catalyst by pH_2_ bubbling, 300 μl D_2_O was added to the solution. Parahydrogen was bubbled through the resulting sample (VI) and a ^13^C spectrum was acquired at 1.1 T with the highest SE = 38. It was also found experimentally and confirmed by *T*_1_ measurements with shuttle system (Fig. S8†) that relaxation time for pyruvate at the solution discussed here was lower than for pyruvate in the (IV) solution used in previous experiments.

**Fig. 7:**
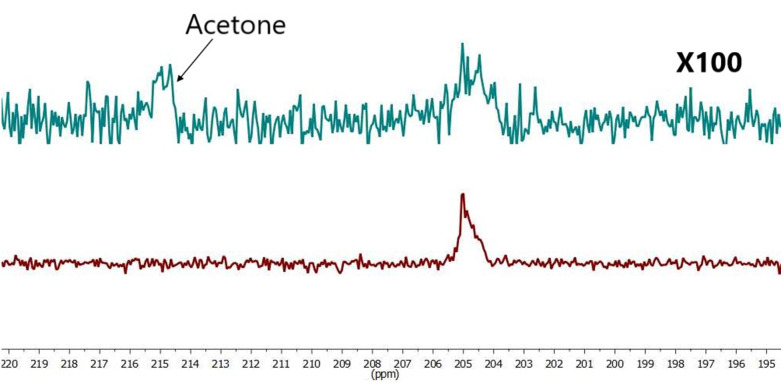
Comparison hyperpolarized (red) and thermal (green, 441 scans ×100 times) spectra of sample VII SE = 56.

For additional experiments the same amount of pyruvate as used in sample (VI), was dissolved in 50 μl D_2_O and [Ir(IMes)(COD)Cl] was dissolved at 100 μl acetone-d_6_. 300 μl D_2_O was added to the solution after continues pH_2_ bubbling during 1–2 min to achieve catalyst activation. The resulting solution formed sample VII. SABRE hyperpolarised ^13^C spectra were acquired (Fig. 7) and a SE = 56 observed for this sample.

As shown in Fig. 7 we have successfully demonstrated pyruvate hyperpolarisation within a 78% aqueous solution. The next step was to determine what is more important to the magnitude of the observed signal enhancement, the quantity of water within the sample or the concentration of the catalyst within the sample.

### 3.4 Effect of catalyst concentration on SABRE enhancement of [2-^13^C]pyruvate

**Fig. 8:**
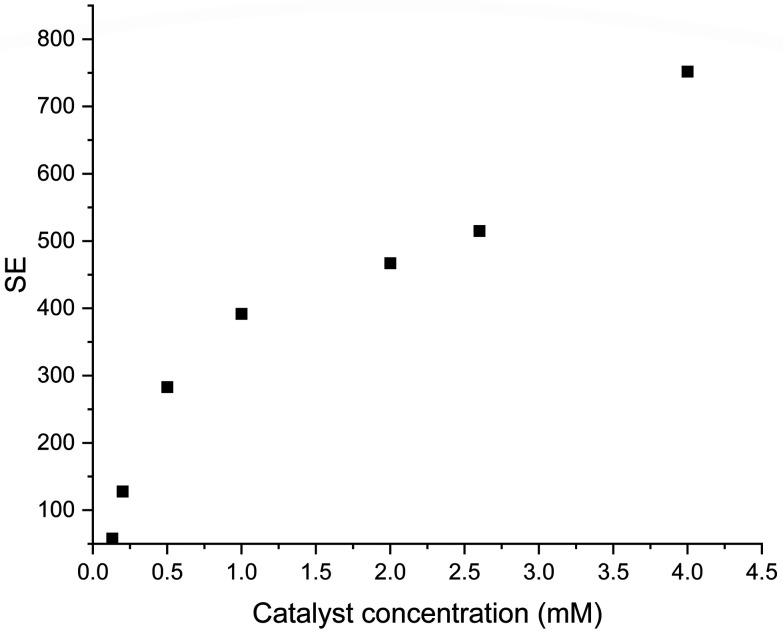
Effect of catalyst concentration on the observed SE for 36 mM [2-^13^C]pyruvate solution similar to sample IV, but with variable catalyst concentration by SABRE.

While the previous section has demonstrated that hyperpolarisation of [2-^13^C] within a predominantly aqueous solution is feasible it is also desirable to decrease the catalyst concentration present for ease of downstream purification. For this purpose the concentration of the catalyst was decreased from 4 mM (IV) down to 0.13 mM (VIII) and the results of this are shown in Fig. 8.

Fig. 8 clearly demonstrates that catalyst concentration has a significant impact on the observed level of hyperpolarisation. Conversely, signal enhancements of two orders of magnitude are still observed when catalyst concentrations are submillimolar. This combination suggests a route towards a biocompatable bolus where catalyst concentration is tuned based on which clean up steps are planned and the desired level of signal enhancement.

**Fig. 9:**
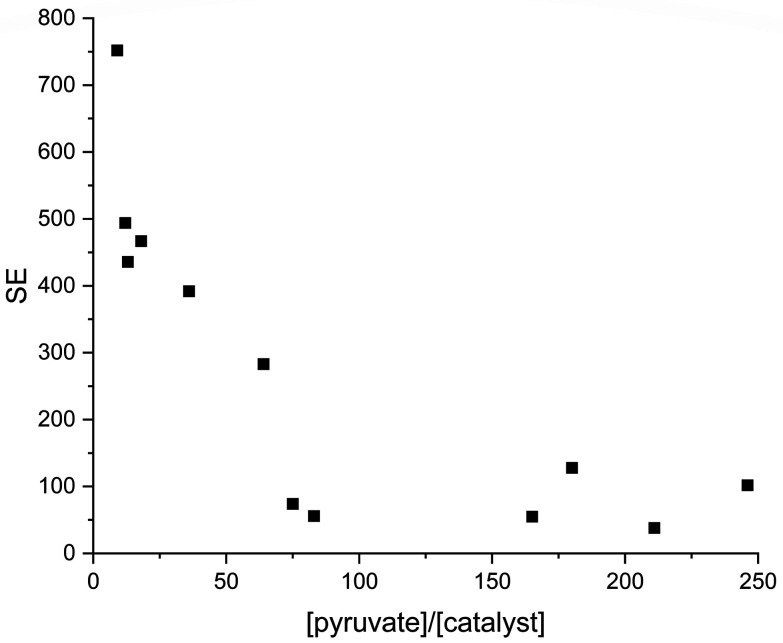
Dependence of SE from [pyruvate]/[catalyst] at different conditions (various of catalyst and pyruvate concentrations), as well as different water/acetone ratio.

To confirm the dependence of SE on catalyst concentration, another series of experiments were carried out. Various concentrations of substrate and catalyst as well as different ratios of water and acetone volumes (Fig. 9) were used (Table S1†). A trend of reduced SE is observed to be proportional to catalyst concentration decrease.

**Fig. 10:**
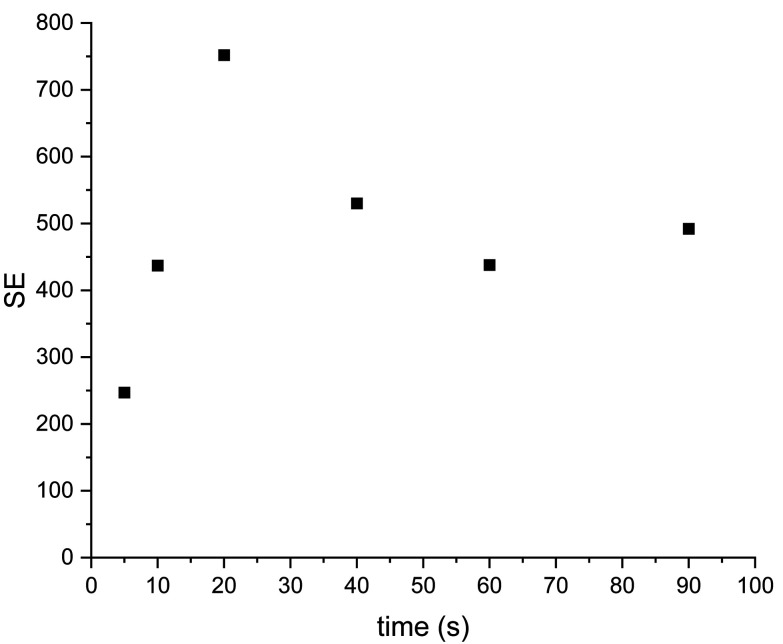
SE dependence from parahydrogen time bubbling for sample IV.

SE dependence from bubbling time for the system (IV) described above were studied to find the best experimental condition (Fig. 10). As can be seen the optimal bubbling time was 20 s for the current system. But for another systems (like samples (V–VIII)) with various catalyst concentration or water amount have been applied also another bubbling times and for some of them longer time up to 120 s of bubbling pH_2_ gave better results (Fig. S10†).

## 4 SABRE of [1-^13^C]pyruvate

**Fig. 11:**
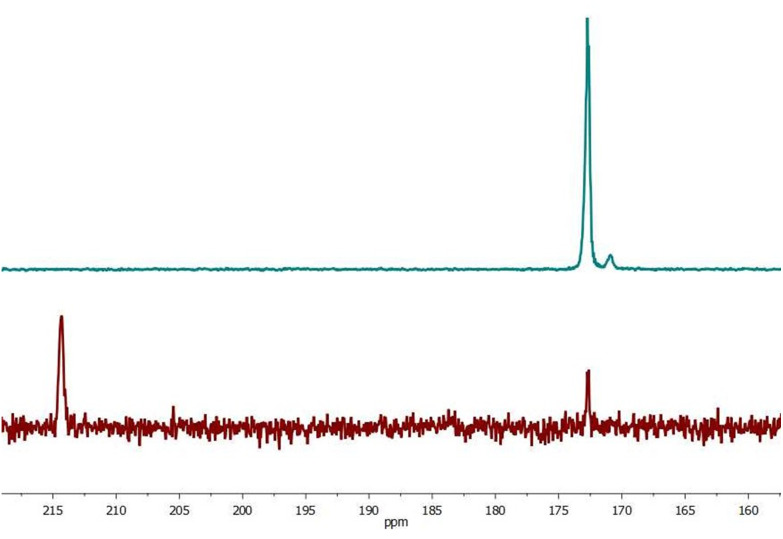
Comparison hyperpolarized (green) and thermal (red, 900 scans ×200 times) spectra for sample II. SE of free pyruvate = 1264, SE of bound pyruvate = 84.

While the primary focus of this work has been [2-^13^C]pyruvate the comparisons with [1-^13^C]pyruvate have been examined. SE found for free [1-^13^C]pyruvate (II) was around twice that of [2-^13^C]pyruvate (IV) under the same conditions as seen in Fig. 11. Should be mentioned that in case of [1-^13^C]pyruvate (II) we could observe signals of both free and bound pyruvate. Another interesting thing is that in case of [1-^13^C]pyruvate the SE dependence from bubbling time appears to be more linear for free pyruvate than that observed for [2-^13^C]pyruvate in Fig. S3.†

## 5 Conclusions

In this paper we outline the advantages a biocompatable bolus of hyperpolarised pyruvate may provide. We have demonstrated SABRE hyperpolarised pyruvate in a predominantly (78%) aqueous solution. This is an important step towards biocompatability as the methanol typically used in SABRE is one of the main sources of toxicity.

Herein, we demonstrate the potential for SABRE in acetone/water mixtures and examine the impact of DMSO as a co-ligand, water quantity and catalyst concentration on the observed signal enhancements. We demonstrate that DMSO increases observed [2-^13^C] SE compared to samples in which it is not present. We demonstrate that higher concentrations of catalyst and lower concentrations of water are beneficial to observed SE. This is shown in sample II where a SE of 1266 ± 34 is obtained for a 33% aqueous mixture containing 4 mM catalyst.

Despite this, herein we aim to demonstrate SABRE of [2-^13^C]pyruvate in a predominantly aqueous solution. Therefore, we report a SE of 53 ± 2 at 1.1 Tesla for a 78% aqueous solution containing 0.7 mM catalyst (sample VII). We report the *T*_1_ of [2-^13^C]pyruvate as 64 seconds at this field of 1.1 Tesla.

This work enables new possibilities for the development of biocompatible SABRE hyperpolarized solutions utilising non-alcoholic solvents. Future work will focus on rapid purification steps to remove residual catalyst and acetone from the hyperpolarised solution, similar to those applied for acetone/water solutions in PHIP.^38^

## Author contributions

O. B.: conceptualization, formal analysis, investigation, methodology, visualization, writing – original draft, writing – review and editing. G. M.: resources, writing – review and editing. T. B. R. R.: formal analysis, supervision, writing – original draft, writing – review and editing.

## Data availability

The data supporting this article have been included as part of the ESI.†

## Conflicts of interest

There are no conflicts to declare.

## Supplementary Material

AN-149-D4AN01005A-s001
