# Supplementary material for: Hyperpolarised [2-13C]-pyruvate by 13C SABRE in an acetone/water mixture
Source: Analyst. 2024 Oct 21;149(23):5668–74. doi: 10.1039/d4an01005a (PMC11529384; doi:10.1039/d4an01005a)
Supplement: AN-149-D4AN01005A-s001 [file AN-149-D4AN01005A-s001.pdf]

Supplementary Information (SI) for Analyst.
This journal is © The Royal Society of Chemistry 2024

Supplementary Information: Hyperpolarised [2-^{13}C]-pyruvate by SABRE in an acetone/water mixture.

Oksana A. Bondar,[1] Gamal A. I. Moustafa,[2] and Thomas B. R. Robertson[1,*]
[1] *School of Chemistry, Highfield Campus, Southampton, SO17 1BJ United Kingdom.*
[2] *ATDBio (now part of Biotage), Highfield Campus, Southampton, SO17 1BJ United Kingdom*

I. TOXICITY

The oral LD50 of acetone varies depending on the animal, and is 5,250 mg/kg in mice pretreated with olive oil, 5800 mg/kg in rats, 3000 mg/kg in mice, and 5340 mg/kg in rabbits.[1]

A potentially lethal dose of methanol is 30 to 240 mL, or 1 gram per kilogram. Ingesting as little as 30 mL of methanol can cause permanent visual damage.[2]

Regarding D_2O used at current study, only higher concentrations (usually > 20% of body weight) can be toxic to animals and animal cells. Also D_2O is used here only on purpose of NMR study, but other than that at in-vivo application normal H_2O can be used.

II. BUBBLING SYSTEM AND EXPERIMENTAL SETUP

The home-made bubbling system was used to purge NMR samples with parahydrogen gas. The schematics of the setup are shown in Fig. S1.

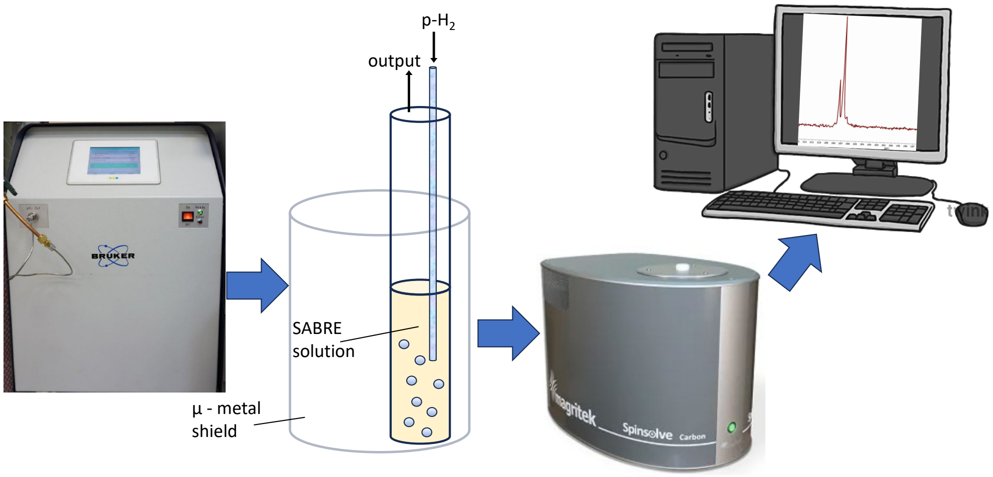

FIG. S1. **Experimental details of the system for SABRE-polarized NMR.**

III. PARA-HYDROGEN ENRICHMENT.

The enrichment is parahydrogen is the first fundamental step to observe PHIP and the hyperpolarizaton level is closely related to the enrichemnt in the para isomer. In our experiment we use a para-hydrogen generator (BPHG Bruker Para-Hydrogen Generator) that operates at 36K and the percentage of para-hydrogen is, nominally, 92%.

---

* t.b.r.robertson@soton.ac.uk

In order to obtain information about possible depleting of para-enrichment, we have measured the p-H_2 percentage immediately after filling the NMR tube with parahydrogen and at increasing time delays, in order to get measurement of the decay rate of p-H_2 in the NMR tubes.

Since p-H_2 is NMR silent, the signal of the ortho- isomer is obsderved. In the Fig. S2 are reported the signals of hydrogen (normal-H_2 and para-enriched) after filling 5mm NMR tube tubes equipped with PTFE gas valves with the hydrogen mixtures, at the same pressure.

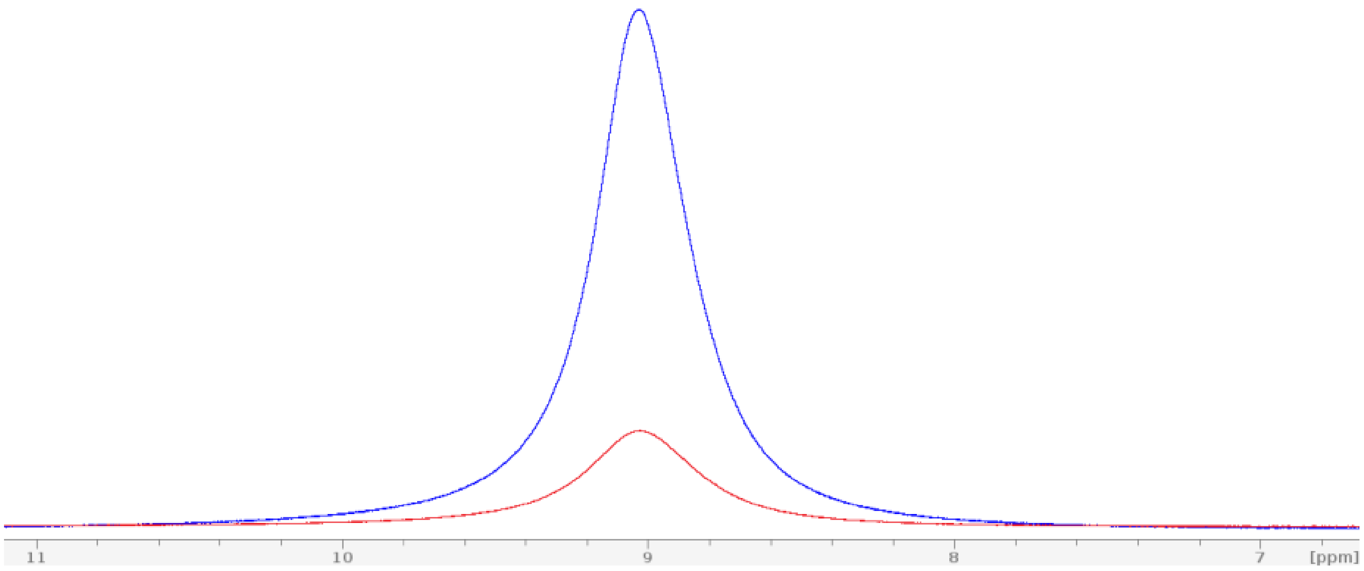

FIG. S2. *o-H_2 signals from a sample of 92% p-H_2 enriched (blue line), from normal-H_2 (red line). In all the experiments the gas pressure was 5000 ± 50mbar, the signal has been precisely normalized to the gas pressure.*

IV. SABRE OF [1-^{13}C]PYRUVATE

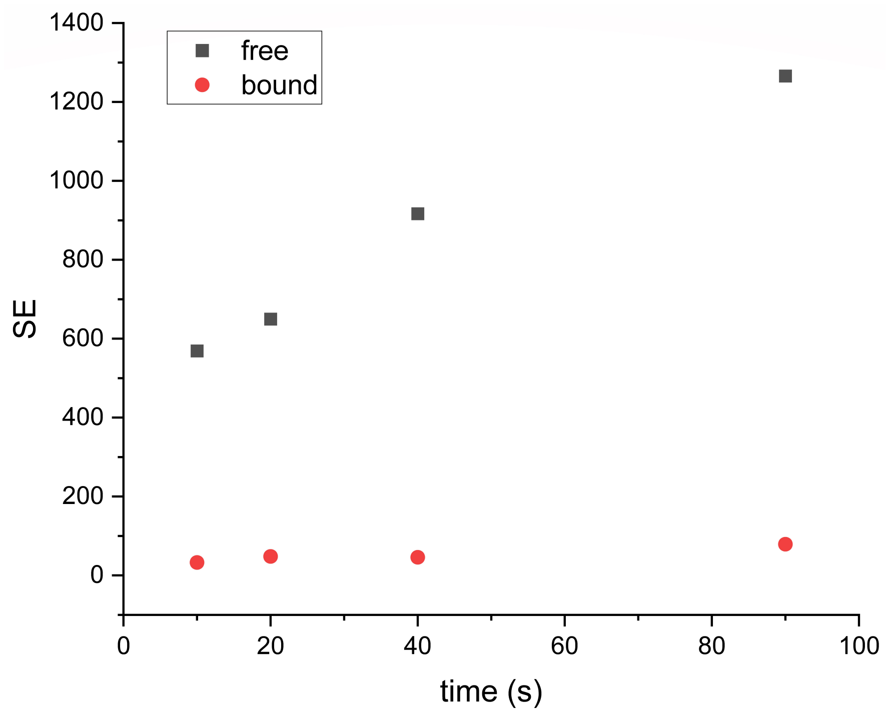

FIG. S3. SE dependence from the bubbling time for hyperpolarized [1-^{13}]pyruvate staying at the benchtop.

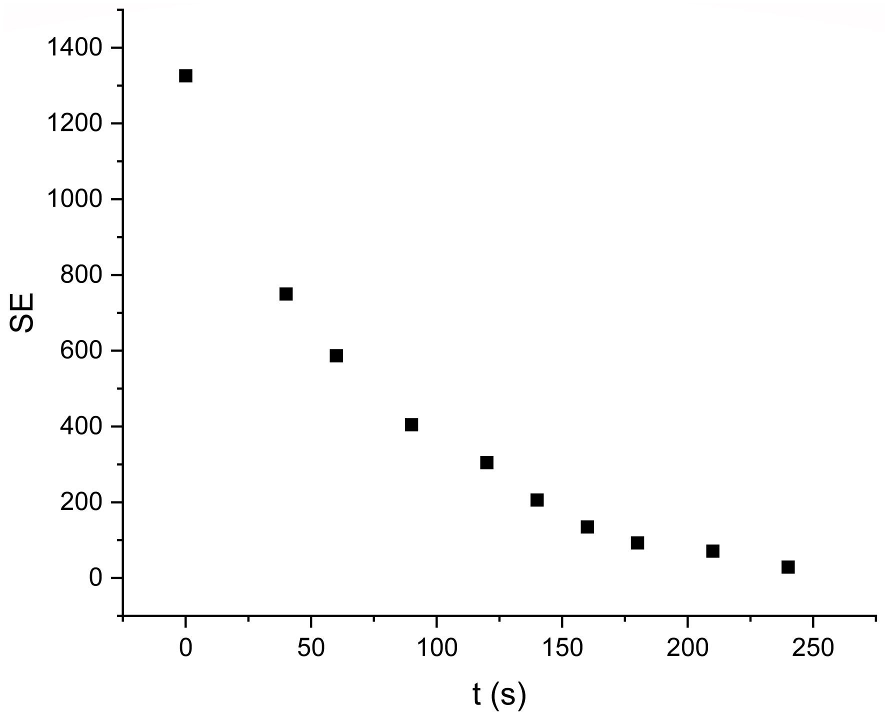

FIG. S4. T_1 decay for hyperpolarized [1-^{13}C]pyruvate measured experimentally.

V. SABRE OF [2-^{13}C]PYRUVATE

TABLE S1. Summary of results from Fig.2 SE dependence for free and bound (Sample I) from bubbling time

Bubbling time	SE(free [2-^{13}C]pyruvate)	SE(bound [2-^{13}C]pyruvate)
5	1133	119
10	1098 ± 174	123 ± 37
20	2148 ± 145	288 ± 61
40	2662 ±142	386 ± 53
60	1937 ± 644	330 ± 176
90	1532 ± 140	187 ± 64
120	2405	357
180	1446	224

TABLE S2. Summary of all samples used to study the effect of catalyst concentration

Number	[pyruvate]/[catalyst]	[DMSO], mM	V(D_2O), μl	V(acetone-d_6), μl	SE
1	9	23	200	400	733 ± 20
2	13	23	200	400	515
3	18	23	200	400	467
4	36	23	200	400	392
5	64	23	200	400	283
6	75	23	100	200	74
7	83	8	350	100	56
8	165	8	100	200	51 ± 5
9	180	23	200	400	128
10	211	4	400	200	33 ± 2
11	246	23	200	400	42 ± 7

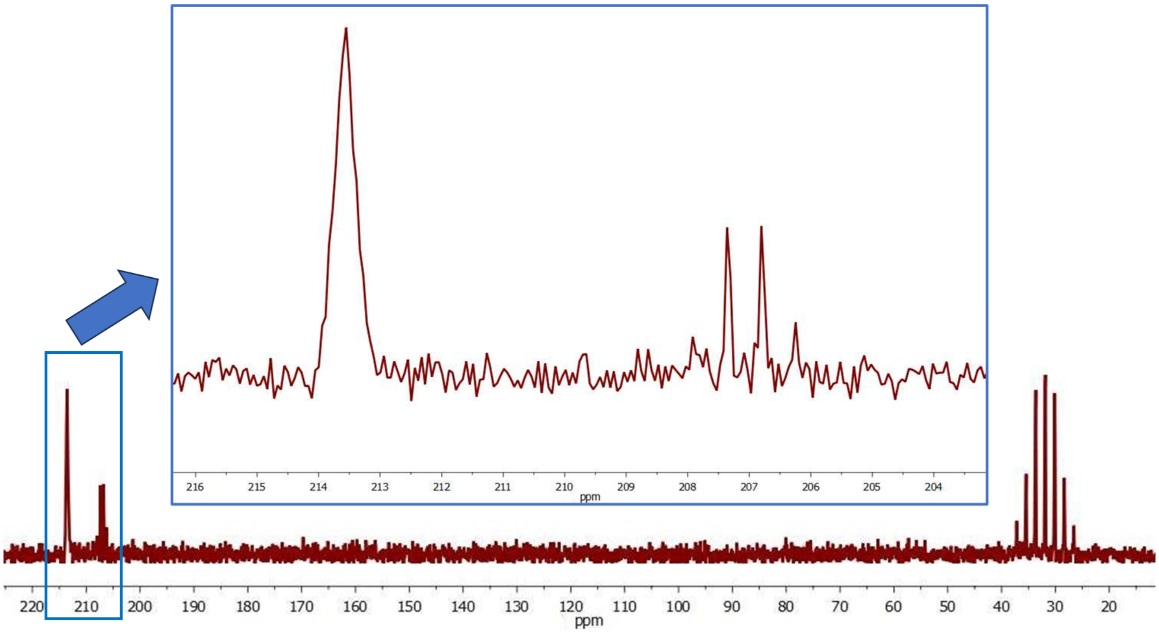

FIG. S5. ^{13}C spectrum of [2-^{13}C]pyruvate in 200 μL D_2O and 400 μL acetone-d_6 without catalyst with acetone signal at around 214 ppm that has been used as external standard to identify free and bounded pyruvate

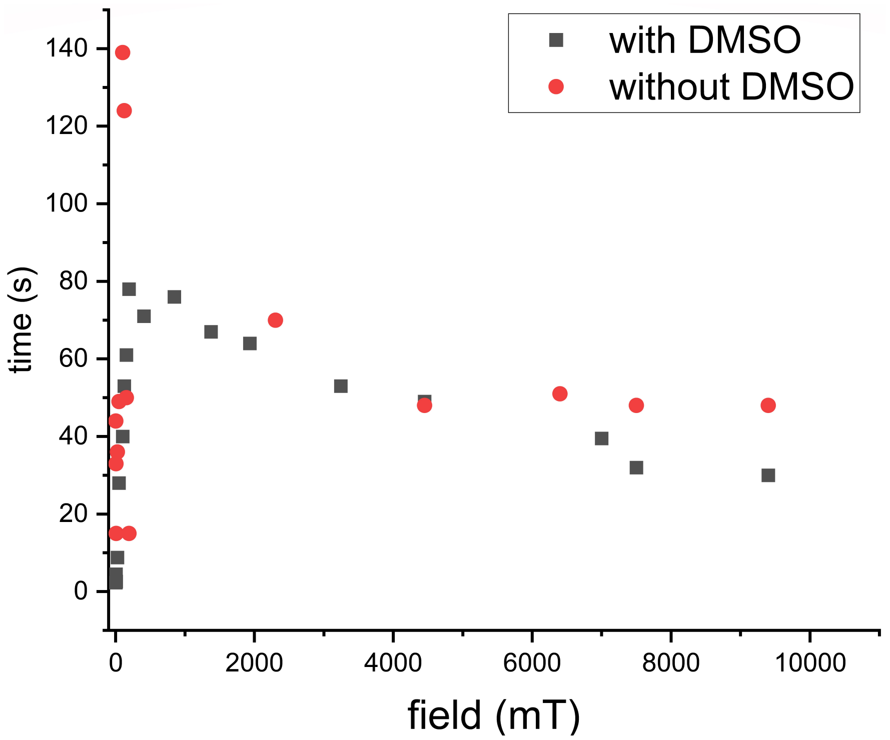

FIG. S6. Comparison of ^{13}C T_1 measurements in different fields using shuttle system for (Sample **III**) and (Sample **IV**)

TABLE S3. Summary of T_1 measurements for Fig.S6

Field, mT	t,s with DMSO	t,s without DMSO
4	2.3	44
6	4.5	33
9.8	2.6	15
26	8.8	36
50	28	49
103	40	139
126	53	124
156	61	50
194	78	15
409	71	
845	76	
1375	67	
1934	64	
2303		70
3243	53	
4450	49	48
6400		51
7000	39.5	
7500	32	48
9400	30	48

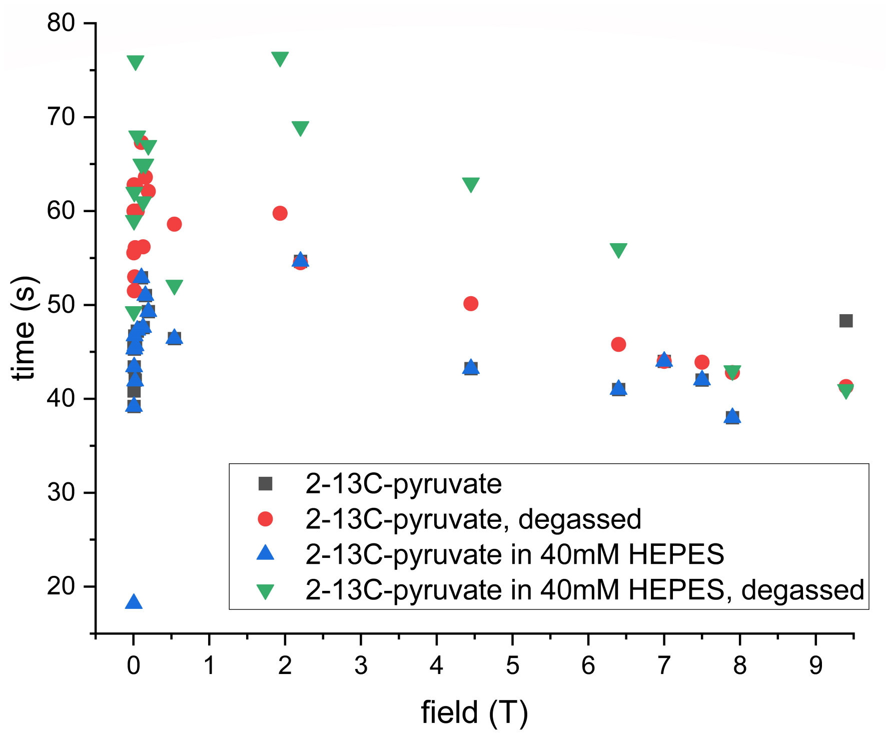

FIG. S7. Comparison of ^{13}C T_1 measurements have been done in different fields using shuttle system[3] for [2-^{13}C]pyruvate in aqueous (pH = 6.83) and HEPES (pH = 6.23) solutions

TABLE S4. Summary of T_1 measurements for Fig.S7

Field, mT	t in H_2O+D_2O t,s	t in H_2O+D_2O, degassed,s	t in HEPES,s	t in HEPES, degassed,s
4	45.6	55.55	18.2	49.3
height 6	39.2	60	39.2	59
8	43.4	62.8	43.4	
9.8	45.3	51.5	45.3	62
14.5	46.7	53	46.7	
26	45.65	62.63	45.65	76
50	47.21	60	47.31	68
103	52.9	67.3	52.9	65
126	47.6	56.2	47.6	61
156	51	63.6	51	65
194	49.3	62.1	49.3	67
1934		59.77		76.4
2203	54.65	54.5	54.65	69
4450	43.22	50.138	43.22	63
6400	41	45.8	41	56
7000	44	44	44	
7500	42	43.9	42	
7900	38	42.8	38	43
9400	48.32	41.3		41

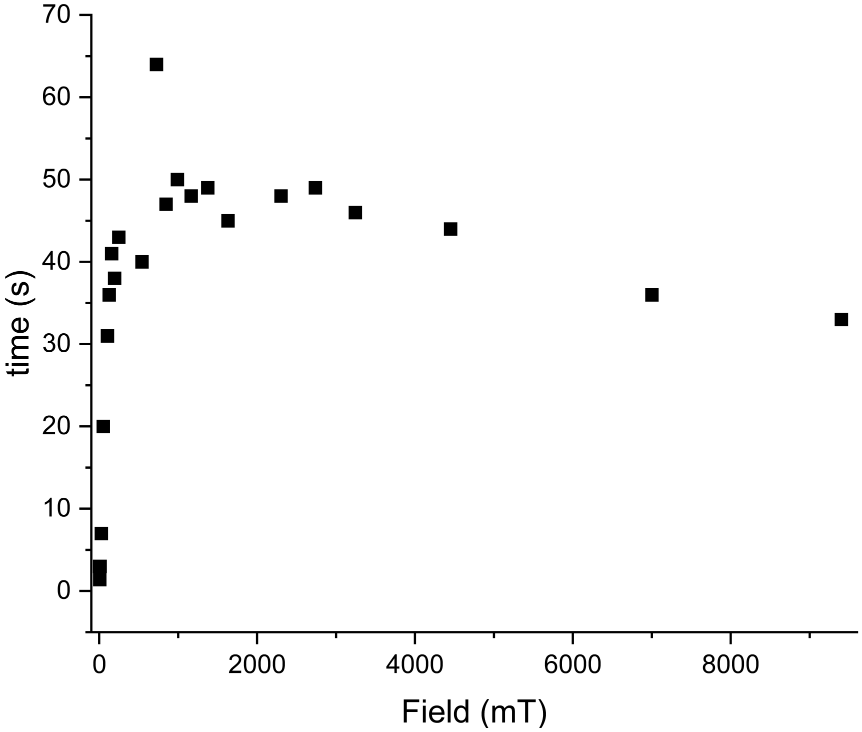

FIG. S8. ^{13}C T_1 measurements for (Sample **VI**) in different fields with final total volume of solution is 600μl

TABLE S5. Summary of T_1 measurements for Fig.S8

Field, mT	t,s
4	3
6	1.4
9.8	3
26	7
50	20
103	31
126	36
156	41
194	38
246	43
540	40
724	64
845	47
990	50
1165	48
1375	49
1630	45
2303	70
3243	48
2737	49
3243	46
4450	44
7000	36
9400	33

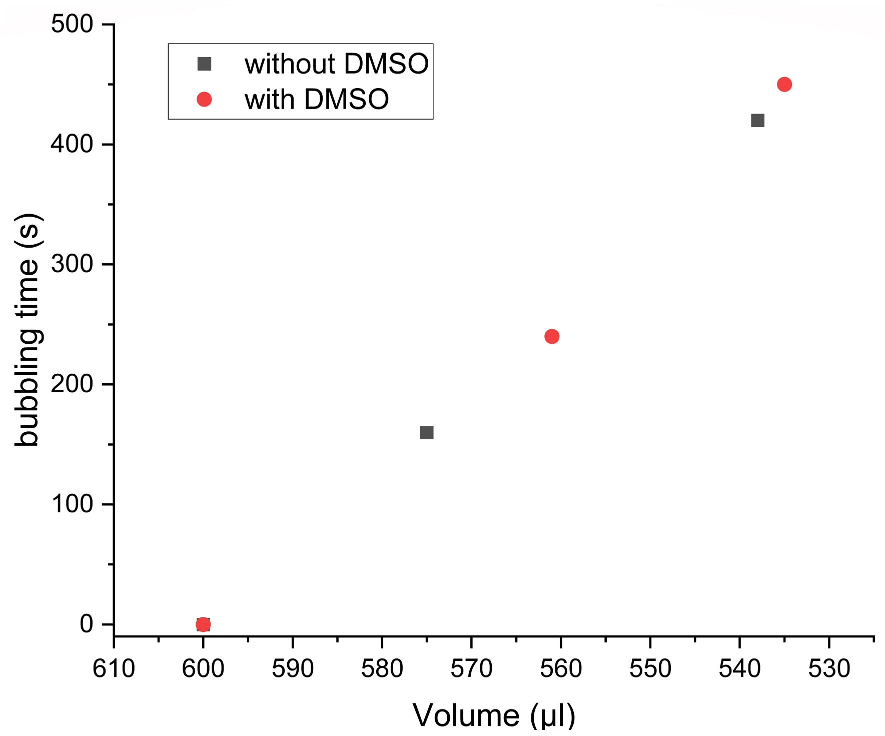

FIG. S9. Volume change over time of p-H_2 bubbling, volume of solution starts from 600μl

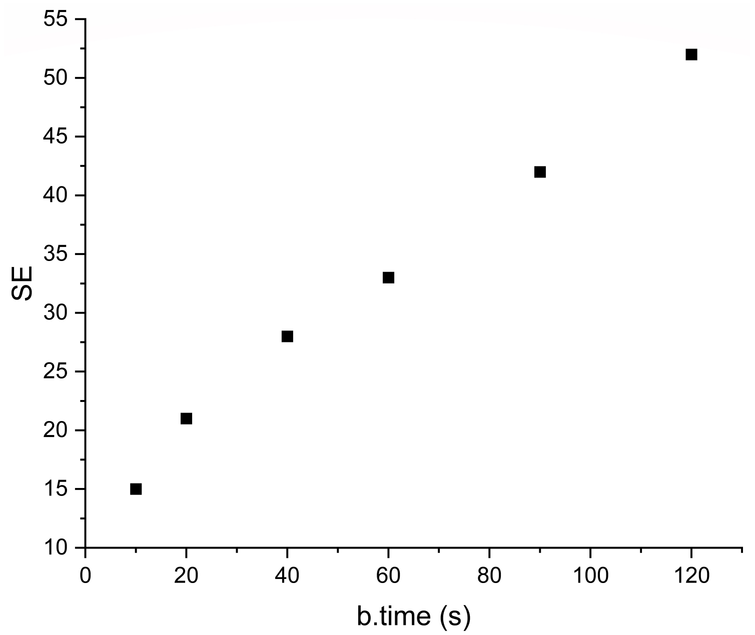

FIG. S10. SE dependence from parahydrogen time bubbling for (Sample **VIII**).

[1] https://www.atsdr.cdc.gov/sites/peer_review/tox_profile_acetone.html.

[2] https://www.ncbi.nlm.nih.gov/books/NBK482121.

[3] C. Bengs, L. Dagys, G. A. Moustafa, J. W. Whipham, M. Sabba, A. S. Kiryutin, K. L. Ivanov, and M. H. Levitt, The Journal of Chemical Physics **155** (2021).
